# Language across the Lifespan in Fragile X Syndrome: Characteristics and Considerations for Assessment

**DOI:** 10.3390/brainsci10040212

**Published:** 2020-04-04

**Authors:** Anne Hoffmann, Angel Wang, Natalie Berger, Lisa Cordeiro, Rebecca Shaffer, Nicole Tartaglia, Craig Erickson, Elizabeth Berry-Kravis

**Affiliations:** 1 Departments of Pediatrics and Communication Disorders, Rush University Medical Center, Chicago, IL 60612, USA; 2 Department of Pediatrics, Rush University Medical Center, Chicago, IL 60612, USA; angel_wang@rush.edu; 3 Department of Psychiatry, Rush University Medical Center, Chicago, IL 60612, USA; natalie_berger@rush.edu; 4 Division of Developmental Pediatrics, Department of Pediatrics, University of Colorado School of Medicine, Aurora, CO 80045, USA; lisa.cordeiro@childrenscolorado.org (L.C.); nicole.tartaglia@childrenscolorado.org (N.T.); 5 Division of Child and Adolescent Psychiatry, Cincinnati Children’s Hospital Medical Center, Cincinnati, OH 45229, USA; Rebecca.Shaffer@cchmc.org (R.S.); Craig.Erickson@cchmc.org (C.E.); 6 Departments of Pediatrics, Neurological Sciences, Biochemistry, Rush University Medical Center, Chicago, IL 60612, USA; elizabeth_berry-kravis@rush.edu

**Keywords:** Fragile X syndrome, language assessment, intellectual disability

## Abstract

While it is widely acknowledged that language development is delayed for the majority of individuals with fragile X syndrome (FXS), there has been limited research into how best to assess this area. This study aimed to deepen the understanding of standardized language assessment in FXS by addressing the three following objectives: (1) Examine the feasibility and validity of widely-used, standardized assessments in participants with FXS; (2) describe linguistic and cognitive profiles for a large sample of individuals with FXS; and (3) Compare results obtained from objective testing in clinic to those obtained using caregiver report. Results indicate that previous results indicating strong correlations between cognition and language results hold true across a wide range of ages as well as across multiple assessments, with an exception in very young children. Caregiver report tended to give lower estimates of language ability than what was found using an objectively administered assessment. Appropriate assessments remain difficult to find as a significant percentage of individuals scored at floor when scaled scores were calculated. Further, a sub-group of participants were coded for behavioral response to testing demands, the majority being able to complete a standardized assessment. These results speak to the need for assessments that provide a wider range of items so individuals can both achieve a valid score and demonstrate progress in their attainment of language skills.

## 1. Introduction

Fragile X syndrome (FXS) is a developmental disorder resulting from an expansion of a CGG repeat sequence in the Fragile X Mental Retardation Gene (*FMR1*) on the X chromosome. This expansion results in methylation and silencing of the gene and subsequently the reduction or absence of Fragile X Mental Retardation Protein (FMRP). FMRP is critical for synaptic function in the brain and overall development and this decrease in its production results in global developmental delay for the majority of individuals with FXS. Males are typically more affected than females, as females benefit from the protective effects of their second X chromosome. With a prevalence of 1 in 4000 for males and 1 in 6000 for females, FXS is the most common form of inherited intellectual disability [1]. Other frequent comorbid behaviors are attention deficits, anxiety, hyperarousal, and autistic symptomatology [2].

### 1.1. Language Development

Language in FXS has been the subject of significant research, although studies have reported conflicting results. While the majority of individuals with FXS will acquire spoken language, initial acquisition of words is often significantly delayed [3]. Brady et al. (2006) found that mothers of 55 young boys with FXS reported them as remaining minimally verbal far longer than what is expected in typical development [4]. It appears that for many individuals with FXS, receptive language development outpaces expressive language, although there is debate as to whether it is commensurate, above, or below nonverbal cognitive development [5,6,7,8].

Within the overall language profile, content has been reported as both a strength and weakness. Hoffmann et al., (2019) found that adolescents and adults with FXS performed better on a receptive vocabulary test than either a nonverbal IQ measure or a global language assessment [9]. Other studies suggest that receptive vocabulary is at the level of nonverbal cognition [5]. Research examining expressive vocabulary has also had varying results, with some early studies showing that expressive vocabulary measures had higher scores than receptive vocabulary measures [6,10] although other studies did not find this discrepancy [11].

As with content, there have been differing results in the area of language form (i.e., syntax and morphology) although it is agreed that this typically falls below chronological age expectations. Several studies have found that receptive grammar falls significantly below nonverbal cognition [3,7,12], while some other research finds that receptive grammar is commensurate with nonverbal mental age [5,11]. Expressively, morphosyntax has been described as comparable to nonverbal cognitive levels [13] in the past, but growing research indicates that it may be area of relative weakness for the majority of individuals with FXS [14].

What is clear from the research is that there is a lack of consensus on the level of impairment for many areas of language in FXS. Many of these discrepancies likely stem from differing age ranges in the samples, the inclusion or exclusion of females with FXS, as well the wide range of abilities inherent in this population. In addition to these difficulties, another source of variation stems from the limited measurement tools available for individuals with intellectual and developmental disabilities (IDD), including those with FXS. The difficulty of assessing language in these populations is discussed below.

### 1.2. Standardized Assessments

When assessing individuals with FXS, researchers and clinicians are faced with the task of finding a reliable instrument that will provide a valid evaluation of that individual’s abilities. The use of norm-referenced assessments theoretically allows for an individual to be compared to their same-aged peers, although the question of whether that individual is actually represented in the norming sample is not guaranteed [15]. For the remainder of this study, the term “standardized” will refer to those norm-referenced, individually-administered, standardized assessments. As individuals with FXS almost invariably have delays in their language development, the majority of standardized assessments in the appropriate age-range do not provide sufficient items at the lower levels to provide a valid standard or scaled score. While growth-scale values are being increasingly provided to allow for growth to be measured when assessments are used out of age range, these scores do not allow for comparisons between assessments, for comparisons to be made with other areas, e.g., cognition, or for comparisons to be made to the normative population. This then forces the use of age-equivalent (AE) scores, which are concerning from a psychometric standpoint as they do not represent an equal interval scale [16]. Furthermore, multiple studies have indicated that standardized testing itself can be a challenging experience for individuals with FXS [17,18]. Given the increased likelihood of attention deficits, hyperarousal, and anxiety, it is unsurprising that the testing process may result in behaviors that make the scores obtained from testing invalid [17,18].

Research into the validity and feasibility of standardized assessment in FXS is limited. Berry-Kravis and colleagues (2013) administered a battery of seven brief standardized tests (total testing time between one and two hours) to individuals with FXS to determine how many were able to complete the assessments as well as if the basic psychometric properties were maintained [19]. They found that none of the tests were able to be completed by all the participants, but that five of the assessments had test-retest ICC or weighted kappa of 0.8 or more, suggesting good reproducibility. At this time, there have been no investigations as to whether longer, more comprehensive, standardized assessments provide valid results when used with individuals with FXS. Accommodations that may help with administration of standardized assessment in general include pairing the initial teaching items with reinforcement, limiting the complexity of verbal instructions, as well as providing frequent breaks and behavioral redirection [20,21]. Research in standardized testing in other populations with ID reveals similar recommendations, e.g., the use of contingent reinforcement with and establishing rapport with individuals with ASD [22,23]. What is well documented is the difficulty of using standardized assessments in populations with IDD, including individuals with a diagnosis of FXS.

Given the difficulty of standardized assessments, clinicians frequently use caregiver report measures to characterize skills. These provide information regarding behaviors and abilities as seen across contexts, with the hope being a more natural representation of the individual’s profile. However, there are concerns when using these as well; they may also ask questions not relevant to characteristics found in FXS, and caregiver report tools are not meant to be used as stand-alone assessments but as a complement to objective measures. Three commonly used caregiver report checklists has been adjusted to better reflect the profile found in FXS [24,25], and it is expected that this research will expand this practice to other tools [19]. Until there are more assessments that provide testing items appropriate to populations with varying intellectual and linguistic capabilities, clinicians must adapt currently available tools to best serve their clients.

This study aims to deepen the understanding of standardized language assessment in FXS by addressing three objectives using widely-available standardized assessments across a wide range of ages and abilities in FXS.

(1) Examine the validity and feasibility of widely used standardized assessments for individuals with FXS, a population with common comorbid diagnoses of intellectual disability, attention deficits, and communication delay.
(2) Describe linguistic and cognitive profiles using cross-sectional data for a large sample of individuals with FXS, providing insight into language use across a wide range of ages as well as potential relationships between language and cognition.
(3) Compare results obtained from objective testing in clinic to those obtained using caregiver report.

## 2. Materials and Methods

### 2.1. Participants

Data analyzed for this study were derived from an extension of the Fragile X Online Registry With Accessible Research Database (FORWARD) using a multisite design (Rush University Medical Center, Chicago; Cincinnati Children’s Hospital Medical Center; University of Colorado/Children’s Hospital Colorado) to collect additional essential longitudinal phenotyping data in individuals with FXS through a comprehensive core battery of outcome measures administered yearly. Data for this study included baseline data from FORWARD—Component C. The study was approved by the Rush University Office of Research Affairs (IRB #: 08121202), Cincinnati Children’s Hospital Medical Center Institutional Review Board (IRB #: 2012-2445), and the Colorado Multiple Institutional Review Board (IRB #: 15-1538). All participants had a confirmed molecular diagnosis of FXS and all participants or their guardians provided written informed consent and participant assent (if feasible) for study participation. Of these participants, 184 had completed at least part of the standardized objective and/or caregiver report assessments and were included in data analysis. Testing was performed at each location by an examiner well-experienced with FXS and standardized assessment in this population.

As detailed below, two assessments were used to assess intellectual ability secondary to the wide age range. For the 24 younger participants who completed cognitive testing standard scores had mean of 73 and standard deviation of 14.0, although five participants scored at floor. For the 142 older participants who completed cognitive testing, standard scores had a mean of 49 and standard deviation of 23.6. The older participants were assessed using the Stanford-Binet, Fifth Edition and the z-derivation formula detailed in Sansone et al. (2014) which expanded the range of scores. This formula has not yet been expanded to other assessments. Without this adjustment, 50 participants would have scored at floor. Participants were also described as to whether they met DSM-V criteria for ASD, as determined by the medical director of their respective FXS clinic. Of the 184 participants, 39 were rated as currently meeting criteria for ASD, 95 were rated as not meeting criteria, and 50 were either rates as unclear or were missing from this rating.

### 2.2. Measures

#### 2.2.1. Bayley Scales of Infant Development—Third Edition

The Bayley Scales of Infant and Toddler—Third Edition (BSID-III; [26]) was used to assess cognition and language abilities for participants 0; 1–3; 6 years of age; as well as those older individuals who were not able to complete testing using one of the assessments meant for older participants. This individually administered, standardized assessment provides separate scores for cognition, receptive language, and expressive language. These areas are assessed using a combination of direct observation of early play skills, as well as elicitation of specific skills for later developing abilities. For the cognition subtest, participants participated in activities ranging from attempting to open a bottle by turning the lid, to completing puzzles. Receptive language included such skills as response to name, pointing to different pictured items, and following multi-step directions. Expressive language included babbling using consonant–vowel sequences, first words, and early syntactic and morphological structures.

#### 2.2.2. Stanford-Binet Intelligence Scales—Fifth Edition

The Stanford-Binet Intelligence Scales—Fifth Edition (SB5; [27]) was used to assess cognition in participants over 3; 6 years of age. The SB5 is a widely-used measure of intelligence and cognitive abilities. The test uses 10 subtests to measure five weighted factors and consists of both verbal and nonverbal subtests. The five factors tested are knowledge, quantitative reasoning, visual-spatial processing, working memory, and fluid reasoning, yielding a nonverbal, verbal, and combined intelligence quotient (IQ).

#### 2.2.3. Clinical Evaluation of Language Fundamental—Preschool Second Edition

The Clinical Evaluation of Language Fundamental- Preschool Second Edition (CELF-P2; [28]) was administered to participants 3; 5–6; 11 years of age. This omnibus language assessment uses a set of three subtests to generate a Core Language Score meant to characterize overall language ability. There is one receptive language subtest, Sentence Structure, as well as two expressive language subtests, Expressive Vocabulary, and Word Structure. Sentence Structure requires participants to demonstrate comprehension of increasingly complex phrases (e.g., point to “The girl is climbing and the boy is swinging”). Expressive Vocabulary requires participants to name different pictured vocabulary and also assesses some early morphological forms (e.g., “ride” vs. “riding”). Word Structure, another assessment of expressive language, provides cloze statements paired with picture stimuli to assess different morphological forms (e.g., “This boy is standing and this boy is ______” to elicit “sitting”). In addition to these Core Language Subtests, participants were also given Word Classes, in which they are shown a group of three to four pictures and asked to identify the two that are most closely related and then explain how those two are alike.

When comparing CELF-P2 subtests with the general receptive and expressive language subscales from the caregiver report measure, Sentence Structure was used to estimate receptive language skills and Expressive Vocabulary was used to estimate expressive language. The Receptive and Expressive Language Indices were not possible as these composite tests do not provide AE scores.

#### 2.2.4. Clinical Evaluation of Language Fundamentals—Fifth Edition

The Clinical Evaluation of Language Fundamentals—Fifth Edition (CELF-5; [29]) is a comprehensive standardized test of language skills, which was administered to all participants over the age of 6. This global language assessment uses various combinations of subtests based on chronological age to derive a Core Language Score. Receptive language subtests include Sentence Comprehension, Word Classes, Semantic Relations, and Understanding Spoken Paragraphs. Expressive language subtests are Word Structure, Recalling Sentences, and Formulated Sentences.

Sentence Comprehension and Word Classes are identical to what is used for the CELF-P2, with the exception that later items for Word Classes do not have picture stimuli and participants are not asked to describe how word are related. Semantic Relations provides participants with a prompt that is read aloud, they are then given four written descriptors that are also read aloud and asked to choose two that are true. For example, an initial training item tells the test-taker that “A man is bigger than a____” with the four choices being “a house”, “a button”, “a plane”, or “a spoon”. Understanding Spoken Paragraphs has the examiner read paragraphs aloud and then ask the participant different text comprehension questions.

For the expressive language subtests, Word Structure is the same procedure as that subtest on the CELF-P2. Recalling Sentences asks participants to listen to sentences of increasing complexity and then repeat them. Formulated Sentences provides participants with a picture prompt and a target word, then they are asked to create a syntactically and semantically appropriate sentence. For participants aged 7–8 years, the subtests were Sentence Comprehension, Word Structure, Recalling Sentences, and Formulated Sentences. For participants aged 9–12 years, core subtests were Recalling Sentences, Formulated Sentences, Word Classes, and Semantic Relations. Participants 13 years old and older were given Recalling Sentences, Formulated Sentences, Semantic Relations, and Understanding Spoken Paragraphs. All participants were also administered Word Classes, in order to have one receptive language subtest that was consistent across ages.

For comparisons with global receptive and expressive language measures, the subtest Word Classes was used to assess receptive language and Formulated Sentences was used for expressive language. The Receptive and Expressive Language Composites were not possible as these composite scores do not have AE scores.

#### 2.2.5. Vineland Adaptive Behavior Scales—Third Edition

The Vineland Adaptive Behavior Scales—Third Edition (Vineland-III; [30]) was administered as an interview to a parent/caregiver for all Component C participants. The Vineland-III is a valid and reliable measure of a person’s adaptive level of functioning from birth to 90 years of age. It is commonly used in clinical care and research to measure the development and functioning of individuals with and without disabilities, including clinical trials in FXS. The Survey Interview Edition was administered to parents or caregivers using a semi-structured interview format. The Vineland-III yields composite domain scores as well as several sub-domain scores. For this study, the Receptive Language and Expressive Language subdomain scores were used.

### 2.3. Data Analysis

AE scores were used to describe performance across tests. While AE scores are psychometrically problematic [31], they were necessary for multiple reasons including (1) the patient population extended beyond the norming sample for the CELF-5, making scaled scores impossible to obtain and (2) a significant percentage of the participants scored at or below floor for these assessments, thus scaled and standard scores do not reflect variability in performance. This is a challenge frequently faced in assessment of individuals with ID [32,33], and one that is the subject of on-going research [20]). As growth scale values are subtest specific, they did not permit comparison across assessments and also provide no reference to the normative population. At this time, there are no alternative means for comparing performance across these tests.

Participants were placed in groups based on the assessment used to evaluate their language and cognition. Participants in Group 1 were assessed using the BSID-III for both language and cognition. Participants in Group 2 were assessed using the CELF-P2 for language and the SB-5 for cognition. Participants in Group 3 were assessed using the CELF-5 for language and the SB-5 for cognition.

Analyses were conducted using SPSS version 26. In order to assess relationships between receptive language, expressive language, and cognition, Pearson product moment correlations were calculated. Caregiver measures and standardized assessments were compared using paired sample *t*-tests. Finally, frequency statistics were calculated to determine the number of participants scoring at floor on various assessments.

## 3. Results

### 3.1. Examine the Validity and Feasibility of Widely-Used, Standardized, and Norm-Referenced Assessments with a Population with Common Comorbid Diagnoses of Intellectual Disability and Communication Delay

#### 3.1.1. Validity

To determine the number of participants able to obtain valid scores using the assessment recommended for their chronological age, frequency statistics were calculated to determine how many participants earned a scaled score at floor. Specific patterns are detailed in Table 1, general trends showed that there was an increasing rate of floor effects with age.

#### 3.1.2. Feasibility

In order to determine whether participation or completion of a standardized assessment was feasible, a subset of 40 participants were coded by the administering clinician as to whether the test administration was complete and valid, or if testing was not possible secondary to refusal or extreme behaviors. These 40 participants received this additional coding secondary to the implementation of the coding schema at their testing location. Of the 40 participants, 5 were in Group 1, 8 were in Group 2, and 27 were in Group 3 based on which tests were administered.

In Group 1, the five participants who were assessed using the BSID-III were able to complete testing and were considered to have received valid scores by the clinician. In Group 2, six of the eight participants (75%) were able to complete the evaluation with one refusing and one not completing testing secondary to time constraints. In Group 3, five participants refused to complete testing and one did not have sufficient time, leaving 21 who completed testing.

### 3.2. Describe Linguistic and Cognitive Profiles for a Large Sample of Individuals with FXS, Providing Insight into Language Use across the Lifespan as well as Potential Relationships between Language and Cognition

**Figure 1:**
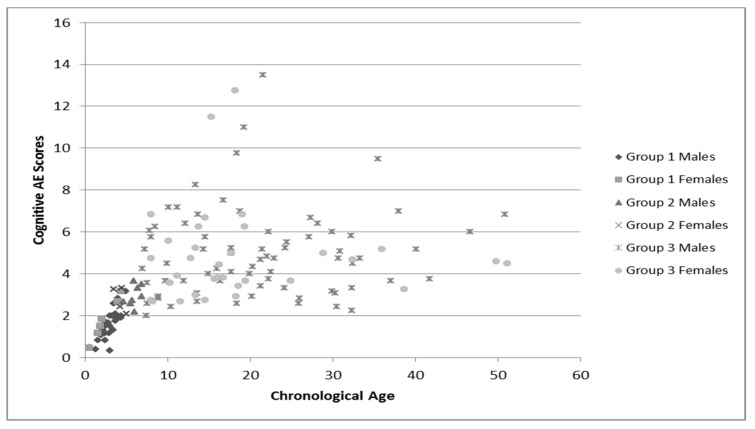
Chronological age and cognitive age equivalent scores. Group 1: Cognition was assessed using BSID-III; Group 2 and Group 3: Cognition was assessed using SB-5.

**Figure 2:**
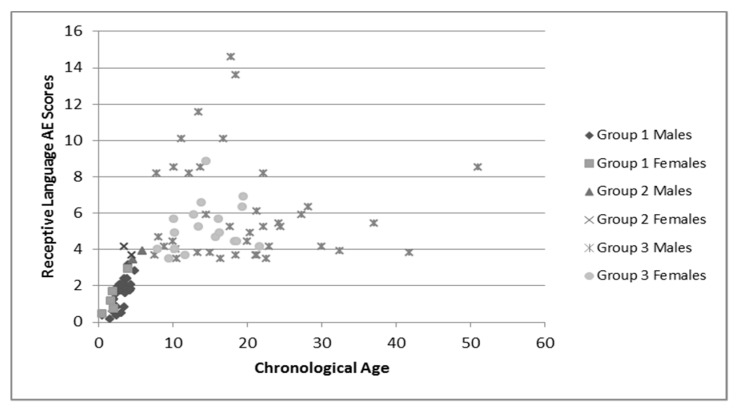
Chronological age and receptive language age equivalent scores. Group 1: Receptive language was assessed using BSID-III; Group 2: Receptive language was assessed using CELF-P2; Group 3: Receptive language was assessed using CELF-5.

**Figure 3:**
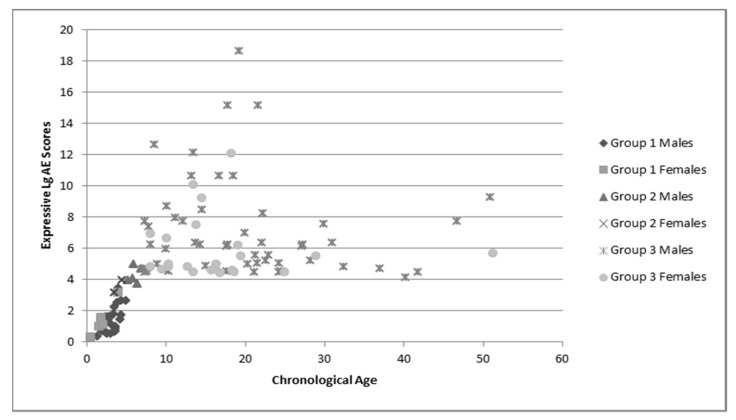
Chronological age and expressive language age equivalent scores. Group 1: Expressive language was assessed using BSID-III; Group 2: Expressive language was assessed using CELF-P2; Group 3: Expressive language was assessed using CELF-5.

Group language and cognitive characteristics are shown in Table 2. Males and females were described separately secondary to the tendency of females to perform better on tests of language and cognition. The assessment recommended for participants’ chronological age was attempted, but if the participant was unable to complete that assessment the one for younger ages was used. Variance in sample numbers are detailed below, this is secondary to participants being unable to either complete all of the assessments (e.g., completed the cognitive portion but not the language portions), or not earning a raw score high enough to have an AE score. Distribution of AE scores for language and cognition by chronological age is shown in Figure 1, Figure 2 and Figure 3.

These figures demonstrate the relatively tight cluster of scores for the younger participants, that become gradually more spread out as the participants become older. This is likely due to the delay that has been well-documented in both cognitive and language development that becomes more apparent as the individuals enter into adolescence and adulthood. At this time, whereas individuals who are neurotypical undergo rapid growth in these areas, individuals with FXS have a much slower rate of growth and some evidence a plateau in development [34]. This is apparent in both the males and females, as the discrepancy between chronological age and age equivalent scores increases with age.

Potential relationships between language and cognition were assessed using Spearman-Rho correlations. These were chosen as the data violated the assumption of normality. As males and females showed the same patterns, they were analyzed together. For Group 1, cognition and receptive language were strongly correlated (*n =* 32, *ρ =* 0.901, *p* < 0.001) as were cognition and expressive language (*n =* 32, *ρ =* 0.798, *p* < 0.001), and receptive and expressive language (*n =* 31, *ρ =* 0.876, *p* < 0.001). In Group 2, cognition was not correlated to receptive language (*n =* 4, *ρ =* 0.400, *p =* 0.600) or expressive language (*n =* 6, *ρ =* 0.130, *p =* 0.805), nor were receptive and expressive language correlated (*n =* 3, *ρ =* −0.456, *p =* 0.699), but the sample size was very small for this group. The participants in Group 3 showed strong correlations between cognition and receptive language (*n =* 51, *ρ =* 0.887, *p* < 0.001), cognition and expressive language (*n =* 71, *ρ =* 0.827, *p <* 0.001), and receptive and expressive language (*n =* 47, *ρ =* 0.817, *p* < 0.001).

### 3.3. Compare Objective and Caregiver Report for Assessment of Language Ability

AE scores from the BSID-III, CELF-P2, and CELF-5 were compared to Vineland-III scores for receptive and expressive language. Paired *t*-tests were used to compare means for the objective test and the caregiver reports. The same subtests were used to estimate receptive and expressive language as detailed above. For these analyses, males and females were combined as both groups demonstrated the same pattern. The only non-significant difference was for expressive language in Group 1. For all other comparisons, caregiver report resulted in an AE score that was significantly lower than the standardized assessment. Further details are shown in Table 3.

## 4. Discussion

This study supports the hypothesis that current standardized language assessments may be inappropriate for individuals with FXS. Given that one purpose of standardized testing is to provide information regarding an individual’s strengths and weaknesses, a significant percentage of participants scored at floor, which precludes this information. This was especially apparent in the older group, in which the percentage of participants receiving the lowest scaled score possible ranged from 33% to 54%. The CELF-5 posed significant challenges for this population, more so than either the BSID-III or CELF-P2. This is almost certainly secondary to too few items in the lower range combined with the significant delays in language common in FXS. Unfortunately, dropping down to the CELF-P2 for those participants unable to achieve a scaled score on the CELF-5 in the hopes of at least calculating an AE score is not always possible as the CELF-P2 requires high raw scores to earn an AE score.

In terms of feasibility, the majority of participants with FXS who were coded for behaviors impacting test administration were able to complete a standardized language assessment, with only 15% refusing to participate. Thus, it seems that most participants are able to participate in standardized assessment, the challenge being to find an appropriate one.

These data support previous findings that individuals with FXS have a high risk of language delay, with findings across a wide range of chronological age as well as across several widely used assessments. As individual’s age increased, there was a wide spread of AE scores, but there was not a linear relationship between chronological age and language ability. The strong correlation between language and cognition in the oldest and youngest groups was expected, this relationship represents the global effects of decreased FMRP on multiple areas of development. The lack of correlation in the middle group is likely reflective of the small sample size, but this area would benefit from further analysis. As this sample includes both males and females with FXS across a wide age range, it appears that this relationship holds true across development and gender.

The discrepancy between standardized assessment and caregiver report for most of the groups was surprising. Except for expressive language in the youngest group, the standardized assessment measured both receptive and expressive language as being more advanced caregiver report did. This was contrary to the expectation that the difficulty inherent in standardized testing with this population would be reflected in decreased scores, whereas the caregiver report could account for performance in more natural settings. The agreement between testing methods for expressive language in Group 1 may represent the clearer milestones early in development that parents may recognize more easily. It is possible that the lack of agreement for the other groups may reflect the limited domains tested by the standardized assessments, whereas the caregiver report covers a wide range of skills and might thus have reflected areas of weakness not found in the others. For example, in Group 3 we used the subtest Word Classes to measure receptive language skills, which is primarily an assessment of semantic knowledge. The discrepancy also speaks to the importance of combining both caregiver and objective assessment in order to gain a complete picture of abilities.

Overall, this study reflects the importance of clinical expertise when choosing assessments for individuals with FXS. The widely used assessments for language may overlook areas of relative strengths and weaknesses, which will make it difficult to determine appropriate intervention targets and determine if progress is being made. Clinicians will likely need to combine several measures to determine an accurate language profile, especially for individuals who, despite being older are still missing foundational language skills.

### Limitations

A primary limitation is the reliance on one subtest on both the CELF-P2 and CELF-5 to estimate expressive and receptive language. However, composite scores for receptive and expressive language were not able to provide AE scores, and given the number of participants who scored at floor as well as those participants who were outside of the age range for the assessments, there were limited options. The variance in sample sizes was also problematic, this was reflective of the challenge many participants experienced in participating in standardized assessment. The small sample that was coded for testing behaviors also limited some of the generalizability of those observations, coding will be extended to all participants going forward.

## 5. Conclusions

The present study aimed to further understanding of language development across the lifespan, as well as the validity and feasibility of widely-used standardized assessments when used with individuals with FXS. Our results suggest that language delays are evident early in development and continue throughout the lifespan for the majority of individuals with FXS. Interestingly, caregiver report characterized language as being lower than the standardized measure scores for the majority of participants. Also, most participants were able to complete the required assessment, but many of them did not achieve valid scores. This lack of appropriate evaluation tools is one that is problematic for all populations with intellectual and developmental disabilities. As clinical trials continue to increase in these populations so does the need for valid outcome measures. Possibilities for alternative assessment tools could include dynamic assessment (in which the assessment task is modified during testing depending on the participant’s abilities) and communication sampling (which provides a more naturalistic view of communication abilities), both of which are appropriate for a wide range of language abilities [34,35]. Future research should continue to explore alternative means of deriving scores, as well as the development of standardized outcome measures that are appropriate for a wide range of language abilities.

## Figures and Tables

**Table 1:** Percentage of participants scoring at floor by subtest.

	Group 1		Group 2		Group 3	
BSID-III	*n*	%	*n*	%	*n*	%
Receptive Language	23	13	---	---	---	---
Expressive Language	23	26	---	---	---	---
CELF-P2						
Sentence Structure	---	---	14	14	---	---
Word Classes			6	83		
Expressive Vocabulary			12	25		
Word Structure			13	31		
CELF-5						
Word Classes	---	---	---	---	41	54
Sentence Comprehension	---	---	---	---	14	36
Semantic Relations	---	---	---	---	51	45
Word Structure	---	---	---	---	12	33
Recalling Sentences	---	---	---	---	64	50
Formulated Sentences	---	---	---	---	48	54

**Table 2:** Participant characteristics.

	Group 1 ^1^		Group 2 ^2^		Group 3 ^3^	
	Males	Females	Males	Females	Males	Females
Chronological Age	*n =* 28	*n =* 6	*n =* 14	*n =* 4	*n =* 90	*n =* 38
M (SD)	3 (1.0)	2.4 (1.5)	5.7 (0.8)	4.3 (0.6)	20.7 (10.7)	18.2 (10.8)
Range	0.5–4.9	0.5–4.3	4.4–6.9	3.5–5.0	6.7–50.9	8–51.2
Cognitive AE	*n =* 28	*n =* 6	*n =* 8	*n =* 4	*n =* 73	*n =* 34
M (SD)	1.6 (0.7)	1.8 (1.0)	2.9 (0.5)	2.77 (0.5)	5.0 (2.1)	4.9 (2.2)
Range	0.3–3.2	0.5–3.2	2.2–3.7	2.1–3.3	2.0–13.5	2.7–12.8
Receptive Language AE	*n =* 27	*n =* 5	*n =* 2	*n =* 2	*n =* 39	*n =* 18
M (SD)	1.6 (0.8)	1.4(1.0)	3.7 (0.4)	3.9 (0.4)	6.1 (2.9)	5.2 (1.4)
Range	0.2–3.2	0.4–2.9	3.4–3.9	3.7–4.2	3.5–14.6	3.5–8.8
Expressive Language AE	*n =* 27	*n =* 5	*n =* 5	*n =* 2	*n =* 54	*n =* 23
M (SD)	1.3 (0.8)	1.4 (1.1)	4.2(0.5)	3.5 (0.6)	6.9 (3.1)	5.9 (2.0)
Range	0.2–3.4	0.3–3.2	3.7–4.9	3.1–3.9	4.1–17.6	4.4–12.1

^1^ Cognition and language were assessed using BSID-III; ^2^ Cognition was assessed using SB-5 and language was assessed using CELF-P2; ^3^ Cognition was assessed using SB-5 and language was assessed using CELF-5.

**Table 3:** Paired T-tests comparing objective testing and caregiver report.

	Group 1		Group 2		Group 3	
Standardized Assessment	BSID-III Receptiv	BSID-III Expressive	CELF-P2 Receptive	CELF-P2 Expressive	CELF-5 Receptive	Celf-5 Expressive
Mean (SD)	1.56 (0.8)	1.4 (0.8)	3.9 (0.3)	4.0 (0.6)	5.4 (2.1)	5.9 (1.9)
Caregiver Report	Vineland-III Receptive	Vineland-III Expressive	Vineland-III Receptive	Vineland-III Expressive	Vineland-III Receptive	Vineland-III Expressive
Mean (SD)	1.3 (0.7)	1.3 (0.8)	2.1 (1.0)	3.1 (0.9)	3.1 (2.4)	4.2 (1.9)
*p*	0.026	0.295	<0.001	0.004	<0.001	<0.001
